# A Users Guide to Holmium Laser Lithotripsy Settings in the Modern Era

**DOI:** 10.3389/fsurg.2019.00048

**Published:** 2019-08-14

**Authors:** Kristian M. Black, Ali H. Aldoukhi, Khurshid R. Ghani

**Affiliations:** ^1^University of Michigan Medical School, Ann Arbor, MI, United States; ^2^Department of Urology, University of Michigan, Ann Arbor, MI, United States

**Keywords:** ureteroscopy, laser, dusting, fragmentation, holmium, moses technology

## Introduction

Endoscopic stone surgery has gone through rapid technological advances in the last decade with the development of next-generation holmium lasers. These systems allow the user to adjust multiple parameters that can optimize fragmentation efficiency during ureteroscopy (URS). Parameters include an increased range in pulse energy (PE), frequency (Fr), and manipulation of the pulse duration (PD). More recently, introduction of pulse modulation with the Moses Technology^TM^ provides pulse modulation with energy delivered over two pulses. In light of these developments, it may be difficult for the urologist to understand how to best utilize laser settings in the modern era. We provide an overview of how PE, Fr, and PD affect three different aspects of laser lithotripsy performance: (A) Fragmentation, (B) Stone retropulsion, and (C) Laser fiber-tip degradation (Table 1).

**Table 1:** Relationship between pulse energy, frequency, pulse duration, and pulse modulation on laser lithotripsy performance parameters.

	**Pulse energy (J)**		**Frequency (Hz)**		**Pulse duration**		**Pulse modulation**	
	**Hi**	**Lo**	**Hi**	**Lo**	**Short**	**Long**	**MC**	**MD**
Fragmentation	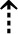	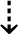	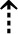	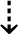	NE	NE	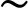	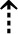
Retropulsion	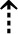	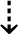	=/ 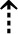	NE	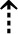	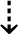	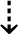	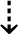
Burnback	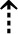	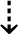	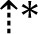	NE	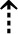	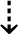	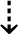	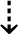

*Hi, High; Lo, Low; NE, no effect; MC, Moses Contact; MD, Moses Distance; ~, comparable effect to both short pulse and long pulse; 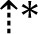, increase in burnback only if total power increases*.

## Pulse Energy

PE is the optical energy emitted from the laser fiber-tip during one pulse and is measured in Joules (J). The PE can vary from 0.2 to 6.0 J and is dependent on the power of the holmium system. Low-power systems (e.g., 20 W) have traditionally had more limited PE power ranges than high-power systems. Factors that influence the selection of PE include stone density, location, and desired fragment size, with commonly utilized settings during URS ranging from 0.2 to 2 J. (A) Fragmentation: Increasing the PE leads to more fragmentation, with hard stones like calcium oxalate monohydrate usually requiring greater PE (1). Higher PE settings such as 1.0 J, are ideal for lithotripsy followed by active basket retrieval. Low PE (LoPE) is employed when utilizing a dusting technique to create very small fragments that are left *in situ* for spontaneous passage. An *in-vitro* study found that using a LoPE setting of 0.2 J leads to the smallest fragment sizes (1) but harder stones such as calcium oxalate monohydrate may require higher PE. In our practice we start dusting settings at 0.2 or 0.3 J and increase the energy accordingly based on how much powder is coming off the stone. (B) Stone retropulsion: On activation of pulsed laser energy, the surrounding fluid evaporates and expands, leading to the formation of a vapor bubble. Collapse of this bubble leads to unwanted movement of stone debris, known as retropulsion. PE has a considerable effect on retropulsion, with higher PEs leading to a proportional increase in retropulsion (1). This decreases fragmentation efficiency and increases procedure time by requiring repositioning of the fiber-tip to maintain contact with the stone, and in the worst-case scenario, a ureteral stone may migrate into the collecting system. (C) Fiber-tip degradation: Higher PE leads to more fiber-tip degradation, also called burnback (2). Degradation of the fiber decreases its length and damages the tip reducing the amount of energy reaching the stone. Contact with the stone can be impaired if the fiber degrades beyond the colored sheath which reduces fragmentation efficiency further.

## Frequency

Fr is the number of optical pulses emitted from the fiber-tip in 1 s expressed in Hertz (Hz). The range available is dependent on the technical constraints of the holmium laser. High-power systems such as the 120 W system can achieve frequencies as high as 80 Hz (3). (A) Fragmentation: Increasing the Fr while keeping PE constant can result in faster fragmentation rates especially when using LoPE settings such as 0.2 J (4), but visibility must also be considered as it can be negatively impacted by higher frequencies. (B) Stone retropulsion: If employing a high PE setting, high Fr will increase retropulsion, and is the reason why fragmentation and retrieval is performed using low Fr. In contrast, when using LoPE, higher frequencies do not have as much impact on retropulsion (4). (C) Fiber-tip degradation: Fr on its own has little impact on burnback, which is more influenced by the total power, especially if using high PE.

## Pulse Duration

PD is the duration of time in which a single optical pulse is emitted measured in microseconds (μs). Conventional holmium systems used fixed PD settings of ~150–350 μs, commonly known as short pulse (SP). Next-generation systems allow for the selection of (SP) or long pulse (LP) modes up to 1,200 μs. LP delivers the same amount of total energy as SP, but over a longer period of time, and has a lower peak power. These differences are exploited to enhance lithotripsy performance. (A) Fragmentation: Overall, there appears to be no significant differences in fragmentation efficiency when utilizing either SP or LP. Most studies have found no significant relationship between PD and ablation volume (5–7) however some have demonstrated more ablation with SP (8, 9). (B) Stone retropulsion: The main advantage of using LP is to decrease retropulsion. *In vitro* studies have reported 30–50% lower retropulsion distances when stones are fragmented with LP compared to SP (8). (C) Fiber-tip degradation: Burnback is also reduced when using LP mode. On average, using LP leads to 5–10 times less burnback than SP (5).

## Pulse Modulation

For SP or LP modes, all the energy is delivered in one pulse causing most of the energy to be lost in vapor channel formation. Pulse modulation is a novel parameter that has recently been introduced as the Moses Technology^TM^. An initial pulse serves to create the vapor channel while the remaining energy is released in a second pulse. The Moses platform has two settings, Moses Contact (MC), intended for operation at a close distance, and Moses Distance (MD) which is designed for lithotripsy at a distance of 1–2 mm. *In vitro* studies have shown that compared to SP and LP modes, MD mode results in 28% greater fragmentation when placed in *contact* with the stone (6). MD mode also results in significantly more ablation when the fiber to stone distance is at 1 mm distance. Due to these reasons, we recommend using this mode for dusting kidney stones, where constant movement is needed to pulverize stones, as well as for non-contact laser lithotripsy (e.g., pop-dusting). However, due to the extended reach of the MD vapor bubble, when treating ureteral stones, we recommend use of the MC mode to efficiently fragment stones with minimal retropulsion. Our recommendation on using the Moses technology is based on several *in-vitro* studies, but more clinical studies are needed to verify these results.

## Other Considerations: Total Power and Safety

PE and Fr selections influence the total power (J × Hz = Watt) which can have important safety implications. Temperatures can increase to concerning levels when using high-power settings and low irrigation rates. In an *in vivo* porcine model with laser activation in the collecting system utilizing 40 W settings, the mean time to reach threshold of thermal injury was 18 s at a medium (14 mL/min) irrigation rate (10). Temperature rises are mitigated if measures such as intermittent laser firing, cooled irrigation, or higher irrigation rates are incorporated. To prevent injury associated with higher intrarenal temperatures, ureteral access sheaths can be used to increase outflow drainage and consequently increase the irrigation rate. Suction technology that removes heated irrigation fluid from the collecting system presents another potential solution to mitigating thermal injury, and is under development.

## Conclusion

Next-generation holmium lasers provide a range of parameters to create efficient lithotripsy strategies. Fragmentation is most affected by PE, and to a lesser extent Fr when utilizing High PE. Retropulsion and fiber burnback can be mitigated by using LoPE settings especially when using a dusting technique, or by using LP mode. Additionally, total power should be considered when selecting parameters for laser lithotripsy, as high-power increases heat generation and may lead to thermal tissue damage. In the future, automated systems may coordinate irrigation rates, laser firing duration, and total power to provide performance characteristics aligned with safety thresholds.

## Author Contributions

KB and AA contributed to constructing the main text and tables of this manuscript. KG was responsible for editing the draft and approval of the final version.

### Conflict of Interest Statement

KG is a consultant for Boston Scientific and Lumenis; KG has a scientific investigator grant from Boston Scientific. The remaining authors declare that the research was conducted in the absence of any commercial or financial relationships that could be construed as a potential conflict of interest.
